# Eldercare services for people with and without a dementia diagnosis: an analysis of Swedish registry data

**DOI:** 10.1186/s12913-021-06891-6

**Published:** 2021-08-30

**Authors:** Atiqur sm-Rahman, Lars-Christer Hydén, Susanne Kelfve

**Affiliations:** 1 Department of Culture and Society (IKOS), Division Ageing and Social Change (ASC), Linkoping University, Kåkenhus, Rum 5516, 601 74 Norrköping, Sweden; 2 Center for Dementia Research (CEDER), Linkoping University, Linköping, Sweden; 3 Department of Culture and Society (IKOS), Division Social Work (SOCARB), Linkoping University, Linköping, Sweden; 4 Department of Neurobiology, Care Sciences and Society, Aging Research Center, Karolinska Institutet & Stockholm University, Solna, Sweden

**Keywords:** Eldercare, Residential care, Home care, Demographic factors, Dementia, Registry data, Sweden

## Abstract

**Background:**

The growing number of people living with dementia (PlwD) implies an increase in the demand for eldercare services in Sweden like in many other countries. Few studies have analyzed the use of eldercare services for PlwD. The aim of the present study is to investigate the association between demographic factors (age, sex, cohabiting status) and the use of municipal eldercare services (including both home care and residential care) for older adults with dementia compared to older adults without dementia in Sweden.

**Methods:**

This study used several nationwide Swedish registers targeting all individuals aged 65 and above living in Sweden in 2014 and still alive 31st of March 2015 (*n* = 2,004,409). The primary outcomes variables were different types of eldercare service, and all participants were clustered based on age, sex, cohabiting status, and dementia diagnosis. In addition to descriptive statistics, we performed multivariate logistic regression models for binary outcomes and linear regression models for continuous outcomes.

**Results:**

Results showed that (1) older age is a significantly strong predictor for the use of eldercare services, although PlwD start using eldercare at an earlier age compared with people without dementia; (2) women tend to receive more eldercare services than men, especially in older age, although men with dementia who live alone are more likely than women living alone to receive eldercare; (3) having a dementia diagnosis is a strong predictor for receiving eldercare. However, it was also found that a substantial proportion of men and women with dementia did not receive any eldercare services.

**Conclusions:**

We found that people with a dementia diagnosis use more as well as start to use eldercare services at an earlier age than people without dementia. However, further research is needed to investigate why a substantial part of people with a dementia diagnosis does not have any eldercare at all and what the policy implications of this might be.

## Background

Currently, an estimated 94,000 out of 160,000 people living with dementia (PlwD) are living at home in Sweden [1]; half of these do not receive any eldercare [2]. Although several studies have been undertaken to analyze the extent and type of home care for older adults in general [3, 4], the number of studies about eldercare for PlwD in Sweden is relatively few.

Eldercare, also known as aged care or long-term care, refers to the care and fulfillment of social and personal needs that allows older adults to live as independently as possible. Eldercare services may vary greatly among countries and change rapidly [5]. Eldercare differs in terms of financing, e.g., private or public financing, whether it is provided in home or in institutions, and the role of the family in care. Thus, the term “eldercare” throughout this article needs to be comprehended from a Swedish perspective.

The Swedish health and eldercare services for older adults are part of a universal welfare system [6–9]. The local tax-funded eldercare services are organized and provided by the municipalities which are the formal decision-making authority for any kind of care services [10]. Decisions about eldercare services are based on an individual need assessment carried out by a municipal needs-assessor.

The main principle of Swedish eldercare is “equal access according to need” [11, 12]. That is, the terms and conditions are the same for older adults to avail of the eldercare service regardless of sex, economic means, and family resources [13]. The Swedish eldercare is divided into two main forms: residential and home care services. Home care services cover home help and personal care, meals on wheels, day care service, short-term and respite care, and guide service [2, 14]. Besides that, assistive technology is an emerging form of home care service includes safety alarms and walking aids etc. [2, 15]. Depending on individual needs, home care services can be offered around the clock. Residential care, on the other hand, refers to receiving institutional care in a long-term residential setting that includes nursing home care (e.g., medical needs, dementia care, terminal illness) and care for individuals who require assistance beyond the usual scope (e.g., special housing including all home care services) [16, 17]. In the Swedish eldercare PlwD are not defined as a special group and are assessed according to the same standards as other eligible persons. In this study, the term eldercare refers to both home care and residential care services.

Significant differences exist in terms of use of eldercare services by older adults across Europe. In an international comparative study, Knapp et al. [18] reported that the percentage of older adults that receive home care services are 25 % in Denmark, 18 % in Norway, 17 % in Canada, and 15 % in Australia. In Norway, residential care services are more common among older adults compared to other countries [19]. In Germany, family members are generally expected to deliver eldercare services and if unable, the government covers all expenses [20]. In Estonia and Spain, there is a legal obligation to family members to provide eldercare support to older adults including economic security [21] whereas in Sweden and Finland there is no such obligation [22].

Literature on the use of eldercare services by PlwD are relatively sparse. Previous studies often focus on PlwD who for example receive any form of care [23], the use of formal and informal care [24], the societal cost for dementia care [25, 26], the need for and use of formal and informal care [27, 28], and quality of eldercare [29]. In a European cross-country study that assessed the home care services for PlwD more similarities than differences were found among eight countries [30]. The authors further concluded that specialized dementia care and services are sparsely available in Europe in general and that PlwD often received only basic formal care with few adjustments to their specific needs [30]. In Scandinavian countries, the allocation of eldercare services for PlwD are mainly based on individual’s needs assessment which is often independent of income.

There are a few studies about the association between demographic factors and the use of eldercare services in Sweden. Some research has found almost no sex difference regarding home care [31], other research revealed that home care service is more common among older men compared to women [32]. A recent Swedish study showed that women used residential care for a longer period than men before death [27]. Older people living alone got more residential care than the older people cohabiting [31, 33–35]. The level of education also influences the care receiving pattern among older adults in Sweden [36]. Research also showed that the use of home care service is relatively more common among PlwD that are living alone or are resident in rural municipalities compared to PlwD that are cohabiting or are living in urban areas [2, 37, 38]. However, how demographic factors like age, sex, and cohabitation status affect eldercare services for PlwD are fairly under-studied.

### Research aim and questions

The overarching aim of this study is to investigate the association between demographic factors (age, sex, cohabiting status) and the use of municipal eldercare services (including both home care and residential care) for older adults 65 years and older with dementia compared to older adults without dementia in Sweden. Specifically, the following research questions were posed:

I What type of eldercare services are older adults aged 65 and over with and without a dementia diagnosis receiving and is there any difference based on their sex, age and cohabitation status?
II Among older adults aged 65 and over with home care service, how many hours of home help service are those with and without a dementia diagnosis receiving, and does sex, age and cohabitation status influence the range of granted home help hours?

## Methods

### Study population

The data in this study were based on information from several nationwide Swedish registers, individually linked by the unique personal identification number assigned to all Swedish residents. We considered all individuals aged 65 and above living in Sweden in 2014 and still alive 31st of March 2015, resulting in a total sample of 2,004,409 individuals. People with dementia was selected based on who have been diagnosed at a hospital or from specialist care (following ICD10-codes F00-F03 or G30-G32) any time between 1st of January 2006 and 31st of March 2015.

### Data sources

Information on sociodemographic factors was extracted from the LISA (Longitudinal integrated database for health insurance and labour market studies) database [39], a longitudinal register including the total Swedish population, while the level of education comes from the National Register of Education [40], both administered by Statistic Sweden. The date of death comes from the National Cause of Death Register [41] and the date of the dementia diagnosis, as well as the number of nights in hospital, were extracted from the National Patient Registers (in-patient care as well as specialist care) [42], all administered by the Swedish National Board of Health and Welfare (*Socialstyrelsen*). Information about eldercare come from the Social Service Register [43], a register also administered by the National Board of Health and Welfare. This register is a source of updated information about municipality’s decisions on eldercare services. The dataset we have used for this study are not publicly available. We ordered the dataset from National Board of Health and Welfare and Statistics Sweden that was provided in 2016.

### Variable specification

#### Outcome variables

The primary outcome variables were the type of eldercare service registered in March 2015, coded into “no eldercare”, “home care services” or “residential care”. In addition, we calculated the “number of home care service hours” per month for all people with more than 0 h of home care service hours in March 2015.

#### Background variables

The date of the dementia diagnosis was set to the first date where the person is registered with a dementia diagnosis in any of the Patient Registers. We considered all diagnoses until March 2015. Individuals having their diagnosis after March 2015 were considered as not being diagnosed with dementia in this study.

Participants’ age was clustered into seven age groups (65 to 69 years, 70 to 74 years, 75 to 79 years, 80 to 85 years, 86 to 89 years, 90 to 95 years, and 95 + years). Days in hospital nights during the last twelve months, here used as a proxy for health [44], were calculated from 1st of March 2014 and were grouped as < 1 day, 1 to 5 days, 6 to 10 days, 11 to 30 days, 31 to 60 days, and 61 to 365 days. The highest level of education was categorized into three levels as compulsory (primary level up to grade 9), secondary (gymnasium up to grade 12), and tertiary (university level). We also considered the civil status of the participants, classified as cohabiting (married or share household) or living alone, and municipalities, characterized as urban, semi-urban, and rural from December 2014.

### Statistical analysis

First, descriptive analyses were performed in order to provide a description of the socio-demographic characteristics, days in the hospital, and the use of eldercare service as percentages, means, and standard deviations. Home care service hours were calculated by mean and standard deviations with accompanying 25th, 50th (median), and 75th percentiles.

Second, we used multivariate logistic regression models for binary outcomes, presented as odds ratios, and linear regression models for continuous outcomes. As odds ratios are problematic to compare between different models [45], we also calculated the results from the logistic regression models as predicted proportions (with the margins command) for men and women, with and without a dementia diagnosis, presented in diagrams. All statistical analyses were performed in STATA 14 (StataCorp, College Station, TX).

## Results

In the total study population, the number of women was somewhat higher (men 46 %; women 54 %) and women were slightly older than men (see Table 1). Among men and women with a dementia diagnosis, non-cohabiting (living alone) men were younger than non-cohabiting women (80.9 vs. 84.2), while the mean age was almost the same for cohabiting men and cohabiting women.

**Table. 1:** Socio-demographic characteristics among men and women 65 years and older with and without a dementia diagnosis (%)

	Men Cohabiting		Men Living alone		Men	Women Cohabiting		Women Living alone		Women	Total
	No dementia diagnosis	Dementia diagnosis	No dementia diagnosis	Dementia diagnosis	**Total**	No dementia diagnosis	Dementia diagnosis	No dementia diagnosis	Dementia diagnosis	**Total**	
*Age*											
Mean(std)	73.7 (6.7)	79.9 (6.8)	74.8 (7.8)	80.9 (7.7)	**74.2 (7.2)**	72.7 (6.1)	79.1 (6.8)	77.9 (8.7)	84.2 (7.2)	**75.8 (8.1)**	75.0(7.8)
65–69	32.7	8.3	31.4	9.5	**31.8**	36.8	9.5	21.2	3.9	**27.7**	29.6
70–74	28.9	14.1	25.9	13.3	**27.6**	30.1	17.4	20.2	7.3	**24.3**	25.8
75–79	18.5	24.2	16.8	18.7	**17.9**	18.0	24.3	16.9	13.3	**17.3**	17.6
80–84	11.5	26.1	11.7	22.8	**11.8**	9.7	25.9	15.8	22.1	**13.3**	12.6
85–89	6.1	19.7	8.5	21.3	**7.2**	4.2	17.2	14.1	28.7	**10.0**	8.7
90–94	2.0	6.9	4.5	11.5	**3.0**	1.0	5.2	8.7	19.3	**5.5**	4.4
95+	0.3	0.8	1.3	2.8	**0.7**	0.1	0.5	3.1	5.5	**1.8**	1.3
*Years with dementia diagnosis*											
Mean(std)		3.3 (2.5)		3.5 (2.5)			3.5 (2.5)		3.7 (2.5)		
*Days in hospital last 12 month*											
0	95.8	86.4	94.9	82.7	**95.3**	96.6	89.1	95.0	84.8	**95.5**	95.4
1–5 days	2.6	4.8	2.5	5.6	**2.6**	2.1	4.1	2.4	5.1	**2.3**	2.4
6–10 days	0.7	2.7	1.0	3.7	**0.9**	0.6	2.0	1.0	3.6	**0.9**	0.9
11–30 days	0.8	4.6	1.1	5.7	**1.0**	0.6	3.8	1.3	5.1	**1.0**	1.0
31–60 days	0.2	1.2	0.3	1.8	**0.3**	0.1	0.8	0.3	1.2	**0.3**	0.3
61–365 days	0.1	0.3	0.1	0.5	**0.1**	0.1	0.2	0.1	0.3	**0.1**	0.1
*Type of municipality*											
Urban	28.5	36.2	29.7	36.9	**29.1**	28.1	36.9	31.8	42.7	**30.4**	29.8
Semi-urban	39.8	34.8	38.1	34.4	**39.1**	40.0	34.5	38.5	31.9	**39.0**	39.0
Rural	31.8	29.0	32.2	28.6	**31.9**	31.9	28.6	29.7	25.4	**30.6**	31.2
*Education*											
Compulsory	33.4	36.3	40.0	43.5	**35.9**	30.9	38.2	40.2	47.9	**36.2**	36.1
Upper secondary	39.4	37.4	38.4	37.1	**39.0**	41.3	38.5	37.0	34.0	**38.9**	38.9
Tertiary	26.2	24.8	19.9	17.4	**23.9**	27.0	22.1	20.5	15.4	**23.3**	23.6
Missing	1.0	1.5	1.7	2.0	**1.3**	0.8	1.2	2.2	2.7	**1.6**	1.4
n	574,716	9458	331,089	7814	**923,077**	479,538	7033	575,694	19,067	**1,081,332**	2,004,409

The number of years people had lived with a dementia diagnosis did not vary much between women and men, neither if they were cohabiting or not. About 5 % of all women and men without a dementia diagnosis spent at least one night in hospital care during the last twelve months, while hospital care was more common among people with a dementia diagnosis. The highest proportion of hospital nights was found among non-cohabiting men with dementia, where almost 17 % spent at least one night in a hospital during the last twelve months. The corresponding number among women was 15 %.

No significant sex differences were found regarding types of the municipality, except among non-cohabiting people with dementia, where more women than men lived in an urban area (42.7 % vs. 36.9 %), while more men lived in a rural area (28.6 % vs. 25.4 %). A majority of both women and men had completed upper secondary education level or more, but the educational level was lower among non-cohabiting women and men.

**Table. 2:** Eldercare status in March 2015 among men and women 65 years and older with and without a dementia diagnosis (%)

	Men Cohabiting		Men Living alone		Men	Women Cohabiting		Women Living alone		Women	Total
	No dementia diagnosis	Dementia diagnosis	No dementia diagnosis	Dementia diagnosis	**Total**	No dementia diagnosis	Dementia diagnosis	No dementia diagnosis	Dementia diagnosis	**Total**	
**Eldercare**											
No eldercare	94.9	40.8	82.6	17.5	**89.2**	94.7	38.9	72.2	11.8	**80.9**	84.7
Residential care	0.9	26.7	4.8	54.5	**3.0**	0.8	29.6	7.1	60.2	**5.4**	4.3
Home care	4.3	32.5	12.7	28.0	**7.8**	4.5	31.5	20.7	28.0	**13.7**	11.0
n	574,716	9458	331,089	7814	**923,077**	479,538	7033	575,694	19,067	**1,081,332**	2,004,409
**Eldercare (community dwelling)**											
Home care	4.3	44.4	13.4	61.5	**8.0**	4.6	44.8	22.3	70.4	**14.5**	11.5
n	569,840	6933	315,294	3555	**895,622**	475,944	4951	534,776	7589	**1,023,260**	1,918,882
**Hours home care if > 0 h/month**											
Min-Max	0.45–744	1-324	1- 744	1-706	**0.45–744**	0.03–744	1-594	0.05–744	1-706	**0.03–744**	0.03–744
Q1. Median. Q3	1. 3. 26	1. 14. 48	1. 8. 36	10. 38. 71	**1. 7. 34**	1. 3. 23	2. 16. 49	1. 6. 33	11. 41. 76	**1. 6. 34**	1. 6. 34
Mean (std)	20(37)	32(42)	24(37)	47(47)	**24(38)**	19(34)	34(45)	23(37)	51(50)	**24(38)**	24 (38)
n	23,326	2274	40,487	2036	**68,123**	20,720	1829	116,913	5148	**144,610**	212,733

About 95 % of the cohabiting women and men without a dementia diagnosis did not have any kind of eldercare, while those living alone received care to a higher degree (28 % of the women and 17 % of the men) – a noticeable sex difference (see Table 2). Among people living with dementia, a much higher proportion did receive some kind of eldercare, in particular among women. Still, 18 % of the non-cohabiting men and 12 % of the non-cohabiting women did not receive any eldercare, although they were living alone and diagnosed with dementia – again a noticeable sex difference.

A similar sex difference can be noticed in terms of residential care: among non-cohabiting individuals, women were more likely to live in residential care compared to men, both among people with (60.2 % vs. 54.5 %) and without dementia (7.1 % vs. 4.8 %). No significant sex differences were found among cohabiting women and men without dementia. However, in the dementia group, the use of residential care was slightly higher among cohabiting women than cohabiting men (29.6 % vs. 26.7 %).

More or less identical sex differences were found for home care services among community-dwelling people, that is, among non-cohabiting individuals, women were more likely to receive home care services compared to men, both among people with (70.4 % vs. 61.5 %) and without dementia (22.3 % vs. 13.4 %), while no significant sex differences were found among cohabiting women and men. Neither did the number of home help hours differ significantly between women and men.

However, all descriptive results from Tables 1 and 2 should be interpreted with the notion that both eldercare, cohabitation status, education, and health (hospital nights) is associated with age, and so is sex, since women, in general, live longer than men that is with increasing age the utilization of eldercare increases especially for older women. As expected, the results from the logistic regression models confirmed that age is a significantly strong predictor for any kind of eldercare, also when adjusted for sex, education, health (hospital nights), cohabitation status, region, and the number of years with dementia (see Table 3).

**Table. 3:** Estimated use of eldercare services in March 2015 among men and women 65 years and older in different age groups adjusted for education, hospital nights, cohabiting status, region, and years with dementia

	Eldercare service		Residential care		Home care service		Hours home care	
	No dementia diagnosis	Dementia diagnosis	No dementia diagnosis	Dementia diagnosis	No dementia diagnosis	Dementia diagnosis	No dementia diagnosis	Dementia diagnosis
	*n* = 1,961,037	*n* = 43,372	*n* = 1,961,037	*n* = 43,372	*n* = 1,895,854	*n* = 23,028	*n* = 201,446	*n* = 11,287
	OR	OR	OR	OR	OR	OR	Coef	Coef
*Age and Sex*								
Men 65–69	*ref*	*ref*	*ref*	*ref*	*ref*	*ref*	*ref*	*ref*
Men 70–74	1.72	1.63	1.83	1.50	1.69	1.50	0.60	9.88
Men 75–79	3.48	2.12	3.70	1.83	3.37	1.88	-0.67	1.22
Men 80–84	8.25	3.31	7.57	2.56	8.03	2.75	-3.10	2.97
Men 85–89	2.67	5.44	16.49	3.48	19.50	4.14	-3.35	5.56
Men 90–94	49.36	10.50	32.38	5.58	43.83	6.50	-1.56	10.73
Men 95+	113.54	13.76	60.89	6.66	89.08	7.40	4.51	26.03
Women 65–69	0.96	0.96	0.66	1.00	1.03	0.94	-1.40	0.40
Women 70–74	1.84	1.79	1.34	1.62	1.96	1.62	-1.50	0.24
Women 75–79	4.17	2.42	3.06	2.07	4.39	2.07	-3.76	2.62
Women 80–84	11.00	4.49	7.18	2.94	11.44	3.60	-5.59	5.46
Women 85–89	29.32	7.55	16.19	3.79	29.11	5.54	-4.43	11.06
Women 90–94	73.20	11.91	35.41	5.80	64.06	6.66	0.16	17.71
Women 95+	157.23	16.42	75.13	9.68	107.14	5.68	10.65	38.28
*Education*								
Tertiary	*ref*	*ref*	*ref*	*ref*	*ref*	*ref*	*ref*	*ref*
Compulsory	1.44	1.03	1.58	1.15	1.35	0.96	-0.17	-1.63
Upper secondary	1.24	1.14	1.33	1.10	1.20	0.99	-0.60	-2.75
Not known	1.18	0.63	1.17	0.60	1.17	0.79	21.77	33.41
*Hospital nights*								
0	*ref*	*ref*	*ref*	*ref*	*ref*	*ref*	*ref*	*ref*
5	1.30	1.26	0.78	1.25	1.39	1.16	-2.66	1.11
10	3.14	1.86	1.10	1.02	3.41	2.03	5.08	6.72
30	7.50	3.02	1.64	1.19	7.92	3.13	11.91	18.17
60	17.54	3.36	3.52	1.31	17.22	3.39	18.99	15.55
365	31.62	4.24	6.96	1.89	30.08	3.50	23.17	17.16
*Cohabiting status*								
Cohabiting	*ref*	*ref*	*ref*	*ref*	*ref*	*ref*	*ref*	*ref*
Living alone	3.46	3.27	4.02	3.04	3.11	2.17	3.54	14.51
*Region*								
Urban	*ref*	*ref*	*ref*	*ref*	*ref*	*ref*	*ref*	*ref*
Semi-urban	0.97	0.85	0.90	0.71	0.99	1.00	1.88	4.12
Rural	0.98	0.93	0.97	1.00	0.98	0.92	-1.09	-0.75
*Years with dementia*								
0–1		*ref*		*ref*		*ref*	*ref*	*ref*
2–5		1.66		2.10		1.19		4.65
6–20		2.15		3.42		1.13		9.58
Pseudo R2/Adj R2	0.341	0.167	0.264	0.132	0.305	0.104	0.030	0.080

Compared with the reference category, that is, men 65–69 years old, all other age groups showed higher odds for receiving eldercare, both among women and men. The same was true for both residential care and home care services. Notable is that the age effect seems to be stronger among people without a dementia diagnosis, shown by very high odds ratios in the oldest age groups. Regarding hours of home care services, the patterns are different. While age seems to have a positive effect on the hours of home care services for both women and men with dementia, age seems to be negatively associated with hours of home care services among women and men without dementia. The exception is the oldest age group (95+), where women are estimated to have almost 11 more hours per month compared with men 65–69 years old. The corresponding number for men is 4.5 h.

**Fig. 1:**
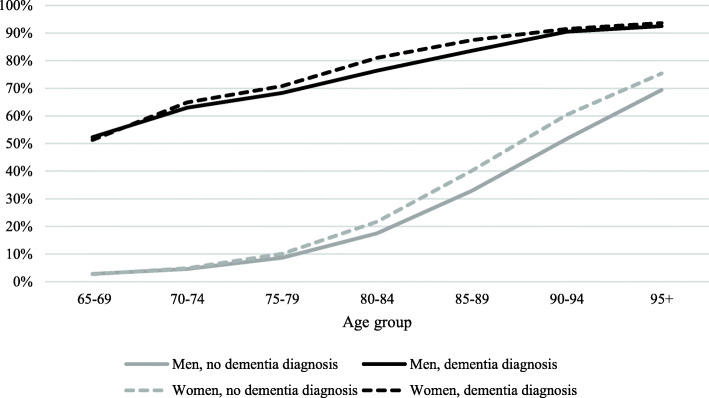
Predicted proportion of receiving any kind of eldercare in all 65 + population by sex, age, and dementia status

The predicted proportions in Fig. 1 confirm that having a dementia diagnosis is a strong predictor for having eldercare. Among both women and men, the predicted proportion of receiving any eldercare at the age of 65–69 years old is about 2.7 % among people without dementia, compared with more than 51 % among people with dementia. For each older age group, the predicted proportion increases in both groups, but more for people without dementia, which results in a smaller gap between people with and without dementia in the oldest age groups.

**Fig. 2:**
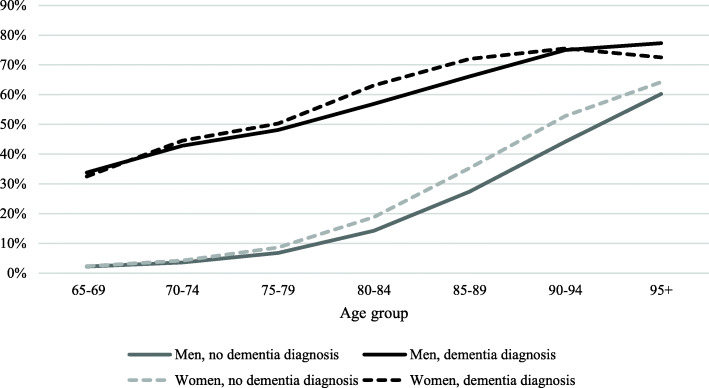
Predicted proportion of home residential care service in all 65 + population by sex, age, and dementia status

**Fig. 3:**
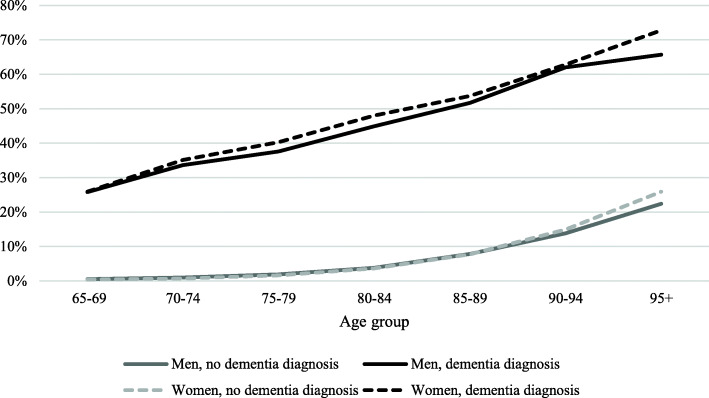
Predicted proportion of receiving residential care in all 65 + population by sex, age, and dementia status

The same pattern of decreasing gap between people with and without dementia is shown for home care services (Fig. 2). In contrast, the predicted proportion for residential care shows a stronger age effect for people with dementia, resulting in a widening gap over age between people with and without dementia (Fig. 3).

For both eldercare and home care services, the sex differences showed the same pattern. Women and men show similar proportions in the younger age groups, while women show a slightly higher proportion in older age groups, in particular from the age of 80–84. The greatest sex differences were found in the age group 85–89 years among people with dementia, where women had six units of percent higher chances of getting home care services than men.

For residential care, few sex differences were observed among people without dementia. For people with dementia, both women and men in the youngest age group showed similar predicted proportions in residential care (26 %), but the difference increases with age. Women in the oldest age group, for example, showed higher proportion of residential care compared to men (73 % vs. 66 %).

## Discussion

In this article, based on Swedish national registers we analyzed the association between different demographic factors and the use of eldercare services among all older adults aged 65 years and over. The findings shed light on the relationships between age, sex, cohabitation status and eldercare services for people with and without dementia. The study identified three differences that are of consequence: (1) the relationship between age and the use of eldercare, (2) sex difference in the use of eldercare, and (3) the relationship between dementia and the use of eldercare. These issues will be discussed.

First, our results show that older age is a significantly strong predictor for any kind of eldercare. In addition, in the younger age groups the proportion women and men that received eldercare services was similar, while a slightly higher proportion of women compared with men received eldercare services in older age groups, in particular from the age of 80–84. A similar finding has been reported by Zielinski and Halling [46] who found that women, especially if aged above 80 years, are more likely to receive home care services compared to men in the same age group. We also found that age is a stronger predictor of receiving eldercare services among people without a dementia diagnosis. This is probably explained by the fact that people living with dementia start to have eldercare earlier and to a higher degree, while people without dementia start to have eldercare later in life. The findings further indicate that age impacts positively on the hours of home care services for people with dementia. This fits with previous findings by Wimo et al. [47], and Bakker et al., [24] who found that older age is associated with more use of care hours in advanced dementia stages.

Second, as already mentioned, sex differences seem to be related to age and older women tend to receive more eldercare than men in accordance with several other studies that have shown that Swedish women receive and use eldercare services to a larger extent than Swedish men [11, 27]. Among people living with dementia, a similar pattern was found: in the age group 85–89 years women had 6 % units higher chances of getting home care services than men. This result corresponds to the findings in a Canadian study [48], although a German study found that home care supports were twice as common for men with dementia than women [49]. Further, it was also found that men with dementia who live alone are more likely than women living alone to have eldercare. A recent study has identified that living alone is the most influential factor in eldercare-receipt for older men who also have less access to formal care services [11].

Third, our study confirmed that having a dementia diagnosis is a strong predictor for having eldercare. That is, people with dementia diagnosis have a higher chance to get eldercare, home care services in particular, compared to people who do not have dementia. Similar findings were found in previous studies where the authors reported that eldercare services were granted to a greater extend to people with cognitive impairment compared to those who did not have a cognitive impairment [30, 34]. This seems to be in line with a European study which showed that access to and use of dementia-specific eldercare services is highly dependent on disease severity along with age, sex, cohabiting status, and region of residence [23]. The number of home help hours and amount of eldercare services for PlwD vary from the time of first diagnosis to the end-of life stage. In other words, as the disease progresses, PlwD will have complex eldercare demands in daily life activities that might need special consideration.

### Strengths and limitations

The main strength of this study is (1) the use of nationwide multiple datasets covering all older adults in Sweden, including those with a dementia diagnosis. (2) We focused on all kinds of eldercare, i.e., both home care and residential care (cf. [30, 34, 36, 50, 51]. (3) We make a comparison between people with and without dementia based on national registry data (cf. [47]).

Our study has some limitations that need to be considered when interpreting the results. First, although the Swedish national health care register is reliable in detecting dementia cases due to its high quality, completeness, and long history [52, 53] some information about individuals living with a dementia diagnosis might be missing. Thus, a main limitation of this study is that we only have data about dementia diagnosis provided by hospital clinics but not about dementia diagnosis provided by the physicians in healthcare centers (*Vårdcentraler*). Second, the cross-sectional design of the study, i.e., where we only consider eldercare services for one month, limits our chances to identify any changes in elderly care use. In addition, using data from only one month also increases the risk for over or under-reporting eldercare service use for some individuals, which could be a result of changes in eldercare services that have not yet been registered. On the other hand, we have no reason to believe that any potential reporting problem should be non-random. Third, information about dementia diagnosis in the National Patient Register (NPR) might be incomplete due to the fact that people in residential care that develop dementia might not be properly diagnoses. Despite this limitation dementia diagnosis in NPR is shown reliable (very high specificity and moderate sensitivity) and the detection rate has improved significantly during the last decades [53].

Finally, like all other studies using these types of register data, we also lack a good measure of health and care needs. Without knowing to what extent, a person is in need of eldercare, we cannot say anything about fairness or inequality in the eldercare provision. Although we added hospital night as a proxy for health, which proved to be strongly correlated with eldercare services, we can only describe differences in eldercare use between groups, not evaluate if these differences are due to actual differences in need or not, that is, if the eldercare system is working as it should. In other words, we did not have independent measurement about needs of care. We discussed in general terms based on the assumption that the older person with dementia probably has greater needs of care compared to the older person without dementia diagnosis.

## Conclusions

We found that people with a dementia diagnosis start to use eldercare services at an earlier age than people without dementia and they also tend to use more eldercare services. However, it is noticeable that a substantial part of people with a dementia diagnosis does not have any eldercare at all, even in the older age groups. This particular group of people need special attention by the research community which can be done by developing explorative research on them. There is also a need for investigating whether the available eldercare services fit with the needs of PlwD.

Finally, our study findings have policy implication as well. In Sweden, the dementia care policy primarily focuses on residential care whereas regulations related to home care services are limited. Our study findings will contribute to the existing gap in dementia care policy in Sweden since there are a considerable number of people living with dementia in their homes without any eldercare at all. We further suggest that more research be conducted to explore why older adults with dementia do not receive eldercare and what are the possible associating predictors of their care outcome.

## Data Availability

The datasets used in the current study are not publicly available, in accordance with specific privacy restrictions. The datasets are stored at the Division Ageing and Social Change at Linköping University. Requests for access to the data that support the findings of this study can be put to the Head of Division, Division Ageing and Social Change, LiU (andreas.motel-klingebiel@liu.se) and will be handled according to the relevant legislation.

## Abbreviations

PlwD: People living with Dementia
LISA: Longitudinal integrated database for health insurance and labour market studies
NPR: National Patient Register
